# Roles of entropic and solvent damping forces in the dynamics of polymer tethered nanoparticles and implications for single molecule sensing†
†Electronic supplementary information (ESI) available. See DOI: 10.1039/c9sc05434k


**DOI:** 10.1039/c9sc05434k

**Published:** 2019-12-11

**Authors:** Guangzhong Ma, Zijian Wan, Hao Zhu, Nongjian Tao

**Affiliations:** a Biodesign Center for Biosensors and Bioelectronics , Arizona State University , Tempe , Arizona 85287 , USA . Email: njtao@asu.edu; b School of Electrical, Computer and Energy Engineering , Arizona State University , Tempe , Arizona 85287 , USA; c State Key Laboratory of Analytical Chemistry for Life Science , School of Chemistry and Chemical Engineering , Nanjing University , Nanjing 210023 , P. R. China

## Abstract

Tethering a particle to a surface with a single molecule allows detection of the molecule and analysis of molecular conformations and interactions. Understanding the dynamics of the system is critical to all applications. Here we present a plasmonic imaging study of two important forces that govern the dynamics. One is entropic force arising from the conformational change of the molecular tether, and the other is solvent damping on the particle and the molecule. We measure the response of the particle by driving it into oscillation with an alternating electric field. By varying the field frequency, we study the dynamics on different time scales. We also vary the type of the tether molecule (DNA and polyethylene glycol), size of the particle, and viscosity of the solvent, and describe the observations with a model. The study allows us to derive a single parameter to predict the relative importance of the entropic and damping forces. The findings provide insights into single molecule studies using not only tethered particles, but also other approaches, including force spectroscopy using atomic force microscopy and nanopores.

## Abstract 2

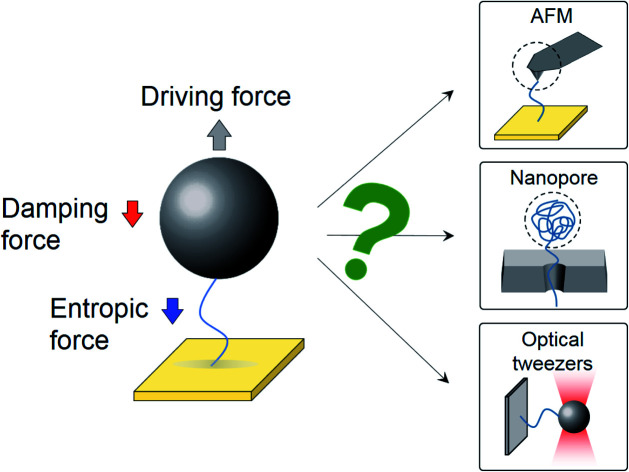
Tethering a particle to a surface with a single molecule allows detection of the molecule and analysis of molecular conformations and interactions.

## Introduction

Detection of single molecules represents an ultimate goal in biosensing and offers a unique capability to study molecular heterogeneity and microscopic processes that are washed out in the traditional ensemble average measurements.^1^,^2^ One successful strategy is to attach one end of a molecule (*e.g.*, a polymer) to a surface, and measure the surface bound molecule with a probe attached to the other end of the molecule. The probe includes the microcantilever probe in atomic force microscopy (AFM), which stretches the molecule while measuring the force.^3^ Another popular probe is a particle,^4^–^8^ which applies a force to the molecule using optical and magnetic tweezers.^7^ A different strategy to detect single molecules is to use nanopores, where a polymer is translocated through the nanopore.^9^ These technologies have provided valuable information on molecules, such as DNA conformation changes,^10^–^12^ DNA–protein interactions,^13^–^15^ and protein–biomarker interactions.^4^,^16^,^17^ Central to all these technologies is the dynamics of the systems, which is controlled by the entropic force associated with conformational changes of the molecule^18^–^20^ and solvent damping force exerted on the molecule and the probe.^8^,^21^–^23^ Despite the importance, the interplay of the two forces and their dependence on the size of the probe, length of the molecule, viscosity of the solvent and time scale of the dynamics remain to be understood.

**Fig. 1:**
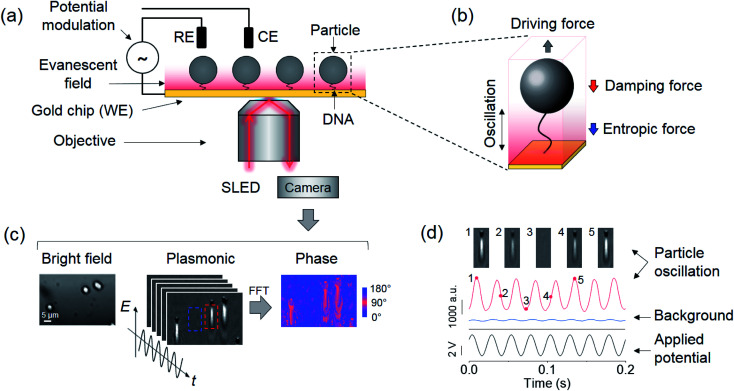
Experimental setup and detection principle. (a) Particles tethered to a gold surface *via* a single polymer and imaged with a plasmonic imaging setup. The particles are driven into oscillation vertically by an alternating electric field applied *via* a three-electrode electrochemical system, where the working (WE), reference (RE), and counter electrodes (CE) are the gold surface, a Ag/AgCl wire, and a Pt coil, respectively. (b) The oscillation of the tethered particle is subject to a viscous damping force by the solvent and an entropic force associated with the conformations of the polymer. (c) Bright field (left), plasmonic (middle) and oscillation phase images (right) of three oscillating particles, where the oscillation phase image is extracted by performing Fast Fourier Transform (FFT) on the recorded plasmonic image sequence over one second. A full video of the oscillation is provided in the ESI.† (d) A particle marked by the red rectangular box in (c) is driven into oscillation (red curve) with an electrical potential with an amplitude of 4 V and a frequency of 40 Hz (black curve), where the particle oscillation is measured from the plasmonic image intensity. Top panels are five snapshots of the oscillation at the marked time points. The blue curve shows the light intensity of an adjacent background region indicated by the blue square in (c). The phase difference between oscillations in particle and background regions is determined as the phase shift of the particle arising from the damping and entropic effects.

Here we study the effects of entropic and damping forces on a particle tethered to a gold surface with a single DNA molecule. We drive the particle into oscillation by applying an alternating electric field to the surface, and study the dynamics of the system on different time scales by varying the frequency of the electric field (Fig. 1a). The oscillation experiences solvent damping and entropic force from the DNA conformation changes (Fig. 1b). We model the dynamics of the particle and the polymer tether by including the solvent damping and DNA conformational entropy effects, and validate the model by tracking the oscillation of the particle with a plasmonic imaging technique and studying the dependence on the frequency of field, type of polymer tether, viscosity of solvent, and size of the particle. We also discuss the implications of the findings for single molecule detection using various platforms.

## Results

### Detection principle

We tethered a streptavidin-coated particle to the gold surface with a double-stranded DNA molecule. The DNA molecule was functionalized with a thiol on one end for attaching to the gold surface, and a biotin on the other end for capturing the particle *via* streptavidin–biotin coupling. The particle was driven into oscillation by applying an alternating electric field perpendicular to the gold surface (Fig. 1a and d) *via* a three-electrode electrochemical system, where the gold surface, a Ag/AgCl wire and a Pt coil served as the working, reference, and counter electrode, respectively. In addition to the electrical force, the particle was subject to an entropic force associated with conformational changes of the DNA tether and damping forces on the particle and DNA from the solvent, which can be described by,1
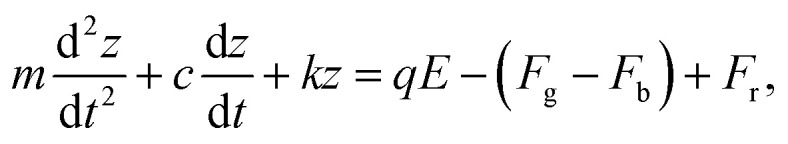
where *m*, *z*, *c*, *q*, *F*_g_, and *F*_b_ are the mass, displacement, damping coefficient, effective charge, gravity and buoyancy of the particle, *k* is the entropic spring constant of the DNA tether, and *E* = *E*_0_ e^*jωt*^ is the electric field applied on the gold surface (*E*_0_ is the field amplitude and *ω* is the angular frequency). *F*_r_ is the stochastic force arising from thermal fluctuations of the DNA and the particle, and has a mean value of zero. The gravity and buoyancy contribute a constant to the *z*, which does not affect the oscillation amplitude at the applied frequency (*ω*). The stochastic force adds Brownian noise to *z* but not the mean value. Simplification of the equation leads to an expression for the phase of the oscillation with respect to the applied electric field, given by (ESI†),2
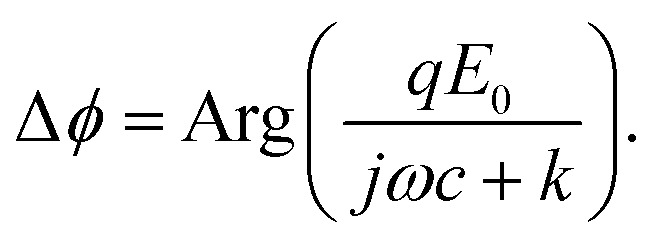



The phase varies from 0° to 90° depending on 
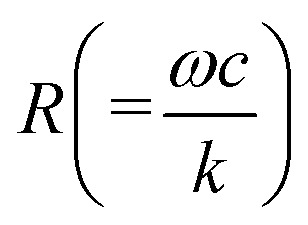
, which measures entropy (*R* ≪ 1) and damping (*R* ≫ 1) dominated regimes.

We used a plasmonic imaging technique to precisely measure the oscillation. The plasmonic setup used a super luminescent emitting diode (SLED) as the light source to excite surface plasmonic waves on the gold surface. The scattering of the plasmonic wave by the particle was imaged with a CMOS camera using an inverted optical microscope (Fig. 1a).^24^ Each particle was resolved as a spot accompanied by a distinct parabolic tail arising from the scattering of propagating surface plasmonic waves by the particle (Fig. 1c). Because the amplitude of the plasmonic wave decays exponentially from the surface to the solution, the image intensity of the particle provides sensitive measurement of the particle–surface distance (thus end-to-end distance of the DNA tether) with sub-nanometer precision (Fig. 1d).^8^ By performing Fast Fourier Transform (FFT) on the image intensity of each pixel in the time domain, the oscillation amplitude and phase were obtained at the frequency of the applied electric field (Fig. 1c), allowing examination of the model described by eqn (2).

### Frequency dependence

**Fig. 2:**
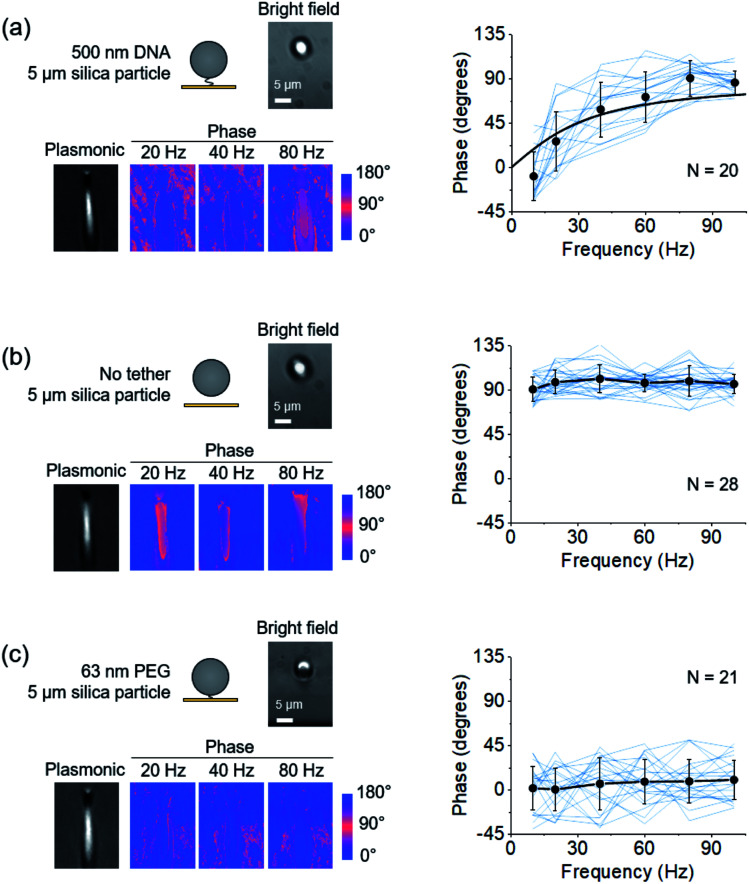
Dependence of the oscillation phase on frequency. (a) Left: A single DNA-tethered particle is driven into oscillation with frequency varying from 10 Hz to 100 Hz. The images are the bright field, plasmonic, and three representative phase images at 20, 40, and 80 Hz of the same particle. Right: The oscillation phase of 20 particles (blue curves) *vs.* frequency. The mean value and standard deviation of phases at different frequencies are indicated by black dots and fitted to eqn (2) (black curve). (b) Left: Bright field, plasmonic, and three representative phase images of the oscillation of a free particle. Right: Phases of 28 free particles (blue curves) *vs.* frequency. The black dots show the mean value and standard deviation at different frequencies. (c) Left: Bright field, plasmonic, and three representative phase images of the oscillation of a particle tethered by a single PEG molecule. Right: Phases of 21 PEG tethered particles (blue curves) *vs.* frequency. The mean value and standard deviation of the 21 measurements are indicated by black dots.

To validate the model, we first studied the dynamics of the system at different time scales by varying the frequency (*f* = *ω*/2π) of the applied electric field. We tethered 5 μm silica particles with 500 nm DNA to the surface, drove the particles into oscillation, and performed FFT on the recorded images to obtain the phase image (Fig. 2a). The phase responses of 20 particles are plotted in Fig. 2a (see Discussion and ESI† for particle-to-particle variability). By fitting the plots with eqn (2), *R* was determined for each particle. *c*, the damping coefficient of the tethered particle, is given by,

3 *c* = 3π*ηD*,

where *η* and *D* are the viscosity of the solvent and the diameter of the particle, respectively. Knowing *c*, *ω*, and *R*, the entropic spring constants (*k*) of the particles were determined to be (8.5 ± 6.9) × 10^–6^ N m^–1^ from the expression of *R* = *ωc*/*k*, which is close to the spring constant of DNA near full extension.^25^ We measured the phase dependence on the frequency of ∼50 DNA tethered particles and found good agreement with eqn (2) (see Discussion).

To demonstrate the importance of entropy in the dynamics of the system, we studied free particles on a gold surface without the DNA tether, drove the particles into oscillation and measured the phase (Fig. 2b). In the absence of the tether, the entropic force drops to zero (*k* = 0 and *R* ≫ 1), and the phase should be ∼90° regardless of frequency. As expected, all of the particles showed a phase of ∼90° (Fig. 2b). As a further demonstration of the importance of entropy, we tethered the particles with a 63 nm polyethylene glycol (PEG) molecule. Because the Kuhn length of PEG is ∼100-fold smaller than that of DNA,^26^ its entropic spring constant is much greater than that of the 500 nm DNA (*R* ≪ 1). The phases of the PEG tethered particles were all close to 0° (Fig. 2c), which was attributed to the high entropy of the PEG tether (ESI†).

### Viscosity and size dependence

**Fig. 3:**
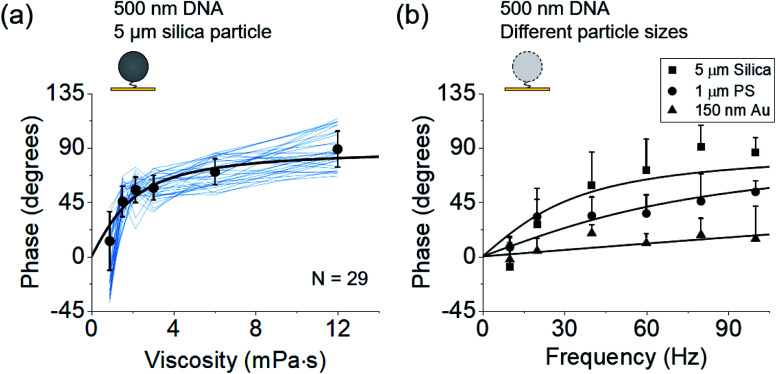
Dependence of phases on the solution viscosity (a) and particle size (b). (a) Phases of 29 DNA tethered particles (blue curves) *vs.* solvent viscosity. The black data points and error bars represent the mean value and standard deviation from 29 measurements, respectively and are fitted to eqn (2) (black curve). (b) Phases of 5 μm silica particles (*N* = 20), 1 μm polystyrene particles (*N* = 17), and 150 nm gold particles (*N* = 14) tethered by a single DNA molecule to the surfaces at different frequencies, where the solid curves are fitting of the data to 
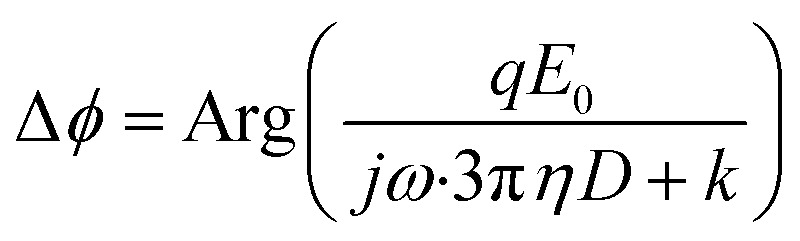
, which is derived from eqn (2) and (3).

According to eqn (2) and (3), the phase of oscillation should also depend on the solvent viscosity. To confirm this prediction, we elevated the viscosity sequentially by introducing tween-20. The phases of individual particles *vs.* the solvent viscosity can be well described with eqn (2) and (3) (Fig. 3a), which provides further validation of the model.

Eqn (2) and (3) also predict that the phase is related to the particle size. To validate this prediction, we tethered 5 μm silica, 1 μm polystyrene, and 150 nm gold particles to gold surfaces using 500 nm DNA molecules as tethers, drove them into oscillation at different frequencies, and measured the phase for each size of the particles (Fig. 3b). Although the particles may have different charges on the surface which lead to different electrical forces (*qE*_0_) and affect the dynamics, the relative magnitude between entropy and damping, determined by 
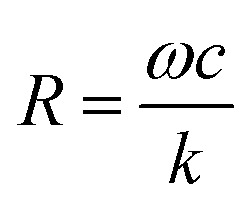
, does not depend on *qE*_0_. The relation between the phase and frequency can be fitted with eqn (2). The phase at the same frequency decreased with particle size due to reduced damping force, which is also expected from the model and eqn (2).

## Discussion

### Single- *vs.* multiple-molecule tethered particles

To ensure that most particles were tethered by a single DNA or PEG molecule, rather than by multiple molecular tethers, the coverages of the tether molecules on the gold surfaces were properly controlled (see Methods). Furthermore, we determined the number of molecules that tethered each particle to the surface by analyzing the motion pattern of the particle, a method established by Visser *et al.*^27^ We examined 516 5 μm particles in total and found that 203 were tethered with single molecules, 217 with two or more tethers, 78 were not tethered, and 18 exhibited switching in the number of tethered molecules (Fig. S1 and S2†). 203 single DNA-tethered particles were used to study the effects of frequency and viscosity on the phase in Fig. 2 and 3 (see ESI† for details). We also measured the phase of oscillation at different frequencies for particles with multiple tethers and observed large fluctuations in the phase (Fig. S3†).

### Phase distribution

We observed a broad distribution in the phase for different particles. For example, the phase histogram of 5 μm particles tethered by single 500 nm DNA molecules shows 10–30° of standard deviation (Fig. S4a†). This distribution could be due to the variation in particle size (10–15%, according to the manufacturer) and the associated influence of volume exclusion effect^28^,^29^ rather than measurement error, which is determined to be 0.47° (ESI†). The effects of particle-to-particle variability on spring constant and *R* value are shown in Fig. S4b.† The dynamic binding and unbinding of DNA to the particle during measurement may also affect the phase (Fig. S2†).

### *R* value criterion

*R* in eqn (2) measures the relative importance of the entropy and damping effects in the dynamics of the system. Using the parameters in our experiments, the *R* value for a double-stranded DNA tethered particle is simplified as4
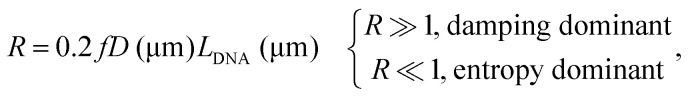
where *f* and *D* are the motion frequency and diameter of the particle, and *L*_DNA_ is the length of the DNA tether in microns. In force spectroscopy (optical and magnetic tweezers) and particle motion analysis, the particle size ranges typically from 50 nm to 1 μm, and the tether length varies from a few tens of nanometers to a few micrometers. Under these conditions and at low frequencies (*f* ∼ 1 Hz), *R* is smaller than 1, which is in the entropy dominated regime. The entropic spring constant for a PEG molecule is ∼100-fold greater than that of a DNA molecule with the same length. Therefore, PEG tethered particles are more likely to be entropy dominant than DNA (ESI†). This finding also indicates that entropy, rather than damping, should be the dominant factor for the nano-oscillators developed by us previously.^8^,^16^,^30^

### Hydrodynamic boundary effects

Driving a particle close to the surface could induce a lateral flow field, which alters the damping coefficient.^31^ When the particle–surface distance (*h*) is much smaller than the particle radius (*a*), the damping coefficient can be determined with *c* = *ac*_0_/*h* (ESI†), where *c*_0_ denotes the damping coefficient when the particle is away from the surface. The phase shift becomes 
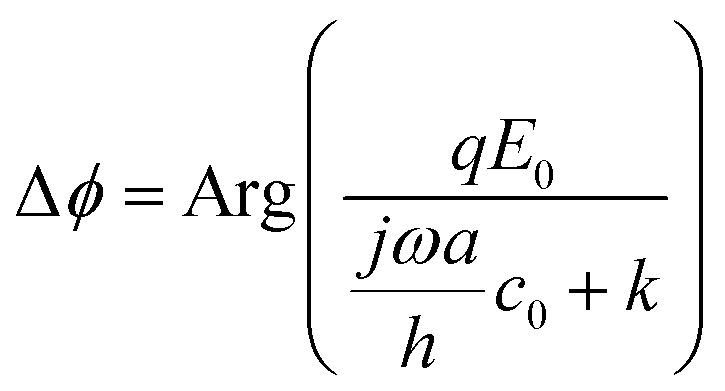
, which takes the same form as eqn (2). This correction does not change the prediction of the transition between entropy and damping dominated regimes. However, numerical fitting of the experimental data with and without this correction produces a large difference in the fitting parameters (ESI†).

### Implication for single molecule sensing

The current work has a significant implication on biosensing using tethered particles. In the solvent damping-dominated regimes, the particle dominates the thermal fluctuations and responds to an external force. For this reason, to study the mobility and size of the particle, it is appropriate to design a system where damping is dominant. In contrast, to probe the mechanical and conformational properties of the single molecule tether, it is appropriate to design the system in the entropy-dominated regime.

**Fig. 4:**
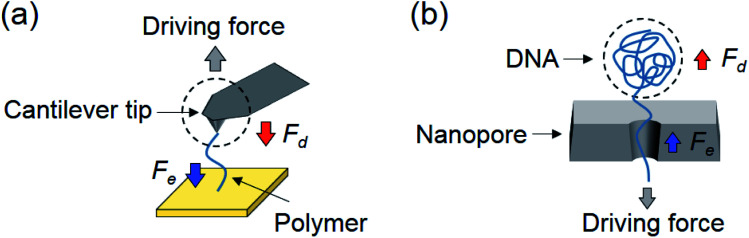
Implication of single molecule sensing. (a) A polymer molecule stretched by an AFM tip to perform force spectroscopy, where the stretched polymer generates an entropic force (*F*_e_), and the movement of the cantilever experiences damping by the solvent (*F*_d_). (b) Translocation of a DNA molecule through a nanopore driven by an electric force and balanced by an entropic force (*F*_e_) and a damping force (*F*_d_) on DNA. The dashed circles in (a) and (b) mark the AFM cantilever and DNA coil, which act like the particle in the tethered particle system.

Although our current experiments focused on tethered particles, we anticipate that they have implications for other detection schemes, including AFM, optical and magnetic tweezers, where entropy from molecular conformations and solvent damping on the molecules and probes affect the dynamics of these systems. Taking AFM as an example (Fig. 4a), for fast force measurement,^32^ one important consideration is the pulling speed of the AFM cantilever because the viscous damping force acting on the cantilever may distort the force spectrum and lead to erroneous conclusions.^23^ It is thus necessary to estimate the magnitude of damping force and compare it with the entropic force. If the damping force is not negligible, strategies should be used to minimize its interference, such as using smaller cantilevers.^33^ Our model in this case also serves as a simple guide to evaluate damping, and determine whether the entropy of the molecule dominates the measurement.

In the case of nanopores, translocation of a polymer also involves a change in entropy (conformation of the polymer) and friction or damping on the polymer by the nanopore and solvent (Fig. 4b).^22^,^34^ For example, the linear section within the nanopore is subject to an entropic force because it is sterically confined,^35^,^36^ and the coiled section outside is subject to solvent damping.^22^ The entropic and damping forces balance the electrical driving force and determine the polymer translocation through the nanopore. At slow translocation, the entropic force inside the pore is the dominant force. However, for fast translocation, the DNA coil outside the pore experiences an increased damping force that can outweigh the entropic force.^22^

## Conclusions

We have studied the dynamics of a particle tethered to a surface with a single polymer, an important system that allows detection and analysis of single molecules. Our work shows that the dynamics of the system is dominated by entropy associated with the conformational changes in the polymer, and the viscous damping of solvent on the particle. Furthermore, it reveals the transition between the entropy and damping dominated regimes, when varying the viscosity of the solvent, size of the particle, type of the tether polymer and frequency of the applied electric field (time scale of the dynamics). The experimental results can be well described with a simple model, where a single parameter can be used to evaluate the relative importance of entropy and damping forces. The findings will help the design and interpretation of single molecule measurements based on tethered particles, including optical and magnetic tweezers, and also other single molecule analysis platforms, such as AFM and nanopores.

## Methods

### Materials

DNA primers were purchased from Integrated DNA Technologies. The Lambda DNA and Phusion High-Fidelity PCR Kit were purchased from New England Biolabs. The Wizard SV Gel and PCR Clean-Up System was purchased form Promega. The spacer molecule, methyl-PEG_4_-thiol [MT(PEG)_4_], was purchased form Thermo Fisher Scientific. SH-PEG10k-biotin, the PEG tether, was purchased form Nanocs. 5 μm streptavidin coated silica particles and 1 μm streptavidin coated polystyrene particles were purchased from Bangs Laboratories. 150 nm streptavidin coated gold nanoparticles were purchased from Nanopartz. Other chemicals were from Sigma-Aldrich. Deionized water with a resistivity of 18.2 MΩ cm^–1^ was used in all the experiments.

### Fabrication of the tethered particles

500 nm double-stranded DNA was obtained by polymerase chain reaction (PCR) amplification of a 1471-bp fragment of lambda DNA using 5′ thiol-modified forward primer (5′ Thiol-GTG TGG ATG CAG CCC TGT T-3′) and 5′ biotin-modified reverse primer (5′ Biotin-TAC GCA GCT CTG CTG TCA CTC-3′). The DNA was separated from the PCR products using the Wizard SV Gel and PCR Clean-Up System. The length of the tether was characterized using gel electrophoresis, and the concentration was measured with a NanoDrop 2000c spectrophotometer (Thermo Scientific).

Gold coated glass slides were rinsed with ethanol and deionized water and then annealed with a hydrogen flame. A polydimethylsiloxane (PDMS) cell was mounted on the glass slide surface for holding the solution. The surface of each gold coated glass slide was partially passivated with 50 μL of MT(PEG)_4_ spacer at a concentration of 1 μM in 1× phosphate buffered saline (PBS) for 10 seconds to minimize the non-specific adsorption of DNA. Then the slide was washed three times with 1× PBS and incubated with 50 μL of the DNA tether at 5 nM in 1× PBS solution for 30 min. Next, the surface was passivated by adding 100 μL of the spacer solution and incubated for 30 min. Finally, the gold chip was washed with 100-fold diluted PBS three times.

Streptavidin coated silica particles were assembled to the distal end of the DNA tether *via* the biotin–streptavidin interaction. 100 μL of the particles at a concentration of ∼5 × 10^5^ ml^–1^ suspended in 100-fold diluted PBS was added to the PDMS in the solution cell and incubated for 30 min. After assembly of the particles to the surface, the solution cell was filled with 150 μL 100-fold diluted PBS and the system was ready for oscillation measurements.

PEG functionalized gold surfaces were prepared using the same protocol. The surfaces with only spacers were prepared by incubating the chip with 100 μL MT(PEG)_4_ spacer at a concentration of 1 μM in 1× PBS for 30 min.

### Experimental setup

The plasmonic imaging setup was built on an inverted microscope (Olympus IX-81) with a 60× (NA = 1.49) oil immersion objective. A SLED (SLD260-HP-TOW-PD-670, Superlum) with a wavelength of 670 nm was used as the light source to excite surface plasmons on the gold surface. A sinusoidal potential was applied by a function generator (33521A, Agilent) *via* a potentiostat (AFCBP1, Pine Instrument Company) and a three-electrode electrochemical setup. The frequency and amplitude of the applied electric potential (*vs.* Ag/AgCl) were 10, 20, 40, 60, 80, and 100 Hz and 0.5, 1, 4, 4, 6, and 6 V, respectively (the currents were ∼1 mA). To reduce the ionic screening effect, the solution used to perform particle oscillation was 100-fold diluted PBS with an ionic strength of 1.5 mM. The oscillation of the tethered particles was recorded by a CMOS camera (ORCA-Flash 4.0, Hamamatsu) at up to 800 frames per second. A USB data acquisition card (NI USB-6251, National Instruments) was used to synchronize the recorded images and the applied potential. More details on the number of particles studied in each experiment, camera frame rate and field of view can be found in the ESI (Table S1†).

### Signal processing

After recording the plasmonic images, Fast Fourier Transform (FFT) was performed on the images collected over one second to extract the phase. A square region of interest (ROI) was selected on the particle and the mean value within the ROI was used to determine the phase of the particle. A clean background region was selected to obtain the phase of the background.

## Conflicts of interest

NJT is a co-founder and collaborator of Biosensing Instrument, Inc.

## Supplementary Material

Supplementary information

Supplementary movie
