# Supplementary material for: Roles of entropic and solvent damping forces in the dynamics of polymer tethered nanoparticles and implications for single molecule sensing
Source: Chem Sci. 2019 Dec 11;11(5):1283–9. doi: 10.1039/c9sc05434k (PMC7747464; doi:10.1039/c9sc05434k)
Supplement: Supplementary file 1 [file SC-011-C9SC05434K-s001.pdf]

Electronic Supplementary Material (ESI) for Chemical Science.
This journal is © The Royal Society of Chemistry 2019

Supporting Information

Roles of entropic and solvent damping forces in the dynamics of polymer tethered nanoparticles and implications for single molecule sensing

Guangzhong Ma[1], Zijian Wan[1,2], Hao Zhu[3], and Nongjian Tao[1,2]*

[1]Biodesign Center for Biosensors and Bioelectronics, Arizona State University, Tempe, Arizona 85287, USA.

[2]School of Electrical, Computer and Energy Engineering, Arizona State University, Tempe, Arizona 85287, USA.

[3]State Key Laboratory of Analytical Chemistry for Life Science, School of Chemistry and Chemical Engineering, Nanjing University, Nanjing 210023, P. R. China

*njtao@asu.edu

Phase of particle oscillation

We estimate the value for each term in the equation of motion of the particle (Eq. 1). The density of 5 μm silica particle is 2.0 g/cm^3. Assuming the oscillation frequency is 20 Hz and the DNA tether can be stretched close to its maximum length (500 nm), the inertia and the damping terms in Eq. 1 are ~4×10^{-4} pN and ~1 pN, respectively. To estimate the entropic force, we use a freely-jointed chain model, which predicts that for a polymer chain comprised of *n* segments of length *b* (Kuhn length) the entropic force (F_e) is given by,

$$F_e = \frac{3k_BT}{nb^2}z \quad , \qquad \text{(S1)}$$

where k_B is the Boltzmann constant, *T* is temperature and *z* is the displacement of the particle. The Kuhn length for DNA and PEG are ~100 nm and 0.76 nm, respectively, from which we obtained entropic forces for a fully stretched 500 nm DNA and a 63 nm PEG, which are ~0.1 pN and ~16 pN, respectively. The inertia term (~4×10^{-4} pN) is much smaller than both the damping and entropic forces, which can be neglected. Also, the stochastic force F_r has a mean value of zero, which contributes to thermal noise in *z*, but does not affect the mean values of *z* and phase shift measured by performing FFT in the present work. Thus Eq. 1 is simplified as,

$$c\frac{dz(t)}{dt} + kz(t) = qE(t) - (F_g - F_b) \quad . \qquad \text{(S2)}$$

The gravity and buoyance of the particle lead to a constant shift z_0 in $z(t)$, which can be described by $z(t) = z_0 + z'(t)$. Thus, Eq. S2 becomes

$$c\frac{dz'(t)}{dt} + kz'(t) + kz_0 = qE(t) - (F_g - F_b) \qquad \text{(S3)}$$

The constant shift z_0 can be determined using the time-independent terms, which is $(F_b-F_g)/k$. The time-dependent terms describe the oscillation of the particle:

$$c\frac{dz'(t)}{dt} + kz'(t) = qE(t) \quad \text{(S4)}$$

For a sinusoidal applied field $E(t) = E_0 e^{j\omega t}$, $z'(t) = z_0' e^{j\omega t}$, which leads to

$$z_0' = \frac{qE_0}{j\omega c + k}. \quad \text{(S5)}$$

From Eq. S5, we obtained Eq. 2 in the main text that describes the oscillation phase in terms of frequency of the driving electric field, viscosity of the solvent, size of the particle and type of the tether polymer.

We also estimate the natural resonance frequency of the system, given by $f_0 = \frac{1}{2\pi}\sqrt{\frac{k}{m}}$, where the spring constant of the tether is $k \sim 1\times10^{-5}$ N/m, and the mass of the particle is $m = 1\times10^{-13}$ kg. Thus, $f_0 \sim 2\times10^{3}$ Hz, which is much greater than the applied frequency.

Hydrodynamic boundary effects on tethered particles

As the particle approaches the surface with a particle-surface distance much smaller than the particle radius, the diffusion coefficient (D) of the particle is affected by hydrodynamic boundary effects, given by,

$$D = \frac{D_0 h}{a}(h < a), \quad \text{(S6)}$$

where D_0 is the diffusion coefficient of the particle when it is far away from the surface, a is the radius of the particle, and h is the particle-surface distance. For a particle with oscillation $z(t)$, the above equation can be written as $D = \frac{D_0(h+z)}{a}$, and the damping coefficient $c = \frac{k_B T a}{D_0(h+z)} = \frac{a}{(h+z)}c_0$, where c_0 denotes the damping coefficient when the particle is away from the surface. For 5 um particles, we found h is ~30 nm and z is ~5 nm. Therefore, $c = \frac{a}{h}c_0$, and the phase shift becomes $\Delta\varphi = Arg(\frac{qE_0}{\frac{j\omega a}{h}c_0 + k})$, which takes the same

form as Eq. 2 except that c is replaced with $c = \frac{a}{h} c_0$. In other words, this correction does not change the prediction of transition between entropy and damping dominated regimes but leads to different fitting parameters. The data in Figure 2a is fitted with the above equation and the spring constant is determined to be $(6.1 \pm 5.1) \times 10^{-4}$ N/m, which is ~50 times greater than the values obtained without considering the boundary effects.

Determination of the number of polymers in each tethered particle

We determined the number of polymers on each particle by tracking the particle pattern following Ref. 13 in main text. In this method, Brownian motion of each particle is recorded in bright field by detecting the particle motion in the absence of external forces for 30 seconds at camera frame rate of 100 frames per second. The motion falls into disc-, stripe-, and dot-shaped patterns, which corresponds to single, double, and multiple DNA tethers (Figures S1a and S1b). Free particles and particles with alternating numbers of tethers were also observed (Figures S1c and S1d).

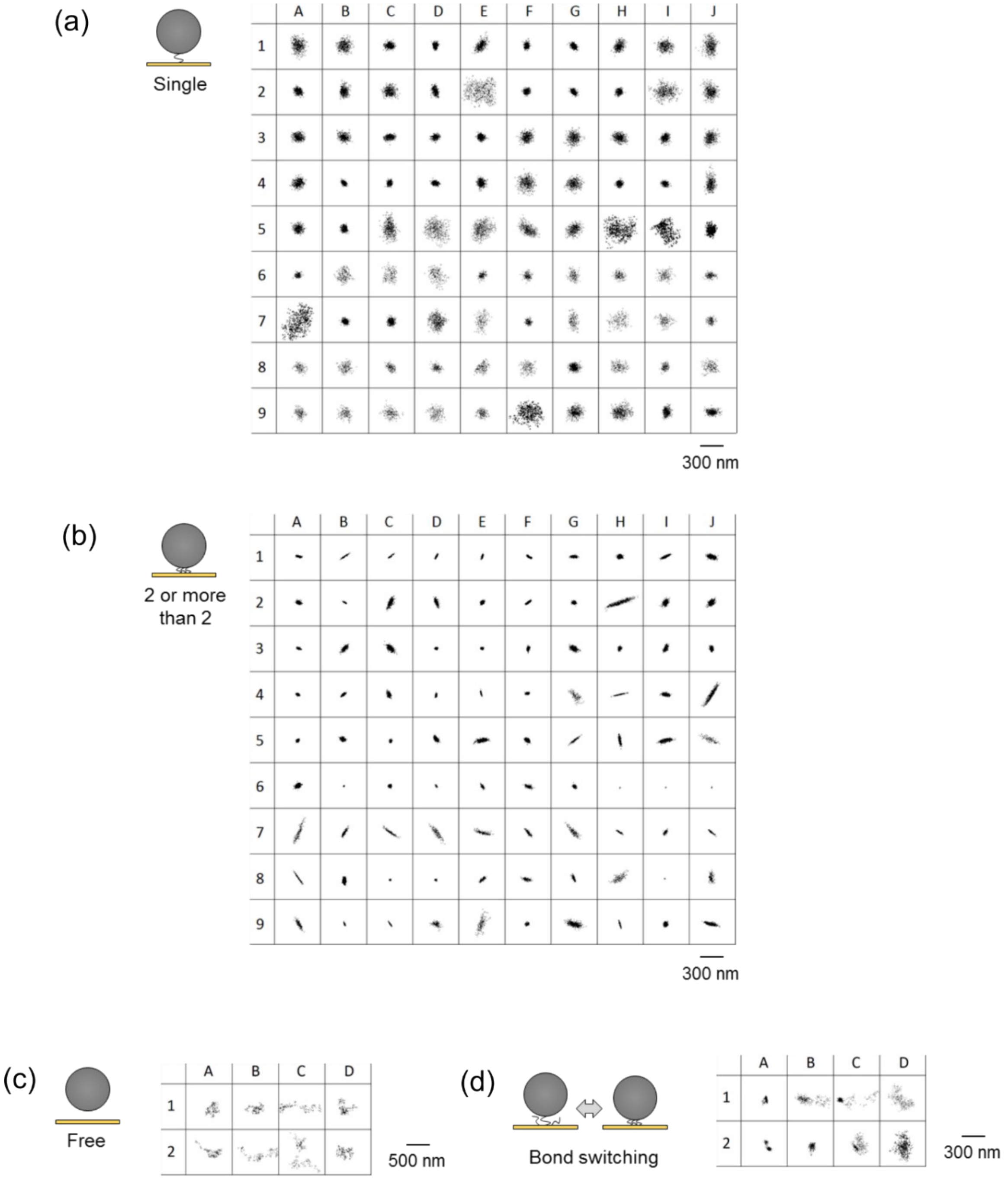

Figure S1. Representative motion patterns of particles tethered by different number of DNA molecules. A total number of 516 of 5 μm silica particles tethered by 500 nm DNA tethers were analyzed. (a) Particles tethered by a single DNA (203 out of 516) showing disc-like patterns. (b) Particles tethered by two or more DNA molecules (217 out of 516) showing stripe- or dot-like patterns. (c) Free particles (78 out

of 516) showing Brownian motion with no confinement by DNA. (d) Particles with fluctuating number of polymer tethers (18 out of 516).

Surface coverage of DNA

Knowing the number of DNA molecules attached to each of the 516 particles, the average density of DNA can be estimated. Assume each of the 5 µm particles occupies a 5 µm × 5 µm region on the surface. Within all the 516 regions, 203 regions have a single DNA molecule, 217 regions have 2 or more DNA molecules, 78 regions have no DNA molecules, and 18 regions have at least one DNA molecule. We estimate the average density by,

$$N_{DNA} = \frac{203 + 217 \times 2 + 18}{516 \times 5\ \mu m \times 5\ \mu m} \sim 5\ DNA/100\ \mu m^2 \quad . \qquad \text{(S7)}$$

Experiments performed with single DNA-tethered particles

After determining the number of DNA tethers in each of the 516 particles, we measured phase responses to different frequencies and viscosities using the 203 single DNA-tethered particles. To capture the oscillation of the particles, the frame rate and field of view of the camera were adjusted with respect to different oscillation frequencies. The details are listed in Table S1.

Among the 203 particles, about 50 particles were used to study the frequency response at 10 Hz with camera frame rate of 200 fps. As we increased the frequency, the frame rate should be increased as well to capture the oscillation of particles. For 20, 40, and 60 Hz, we used frame rate of 400 fps and measured another 50 particles. We further increased the frame rate to 800 fps to measure the response at 80 and 100 Hz, and 50 particles were measured. We did not use the same group of particles for different frequencies because the field of view was changed at different frame rates.

The data obtained as described above were used to construct phase histograms at different frequencies (Figure S4), however, the histograms did not reflect the behavior of the same single particle at different frequencies. It is necessary to examine the phase change of the same particle because the behavior may not follow the statistics. To address this point, we examined the phase of ~25 individual particles at different frequencies (10-100 Hz) using a frame rate of 800 fps, and plotted the phases in Figure 2a.

Another 30 particles were used to study the solution viscosity effect. In the experiment, the phase of each particle was measured at different viscosities. The frequency of the field was 40 Hz and the camera frame rate was 400 fps. The results are shown in Figure 3a.

Table S1. Experiments performed with 5 µm silica particles tethered by a single DNA molecule.

Experiments	Number of particles studied	Frame rate	Field of view
Phase statistics at 10 Hz	~50	200 fps	440 µm × 220 µm
Phase statistics at 20 Hz – 60 Hz	~50	400 fps	440 µm × 110 µm
Phase statistics at 80 Hz – 100 Hz	~50	800 fps	440 µm × 55 µm
Phase response of the same particle at different frequencies (10-100 Hz)	~25	800 fps	440 µm × 55 µm
Phase response of the same particle at different viscosities (frequency = 40 Hz)	~30	400 fps	440 µm × 220 µm
	Total: 203		

Switching of DNA tethers

We observed switching in the DNA tethers in single particles. An example is shown in Figure S2a. The oscillation of a tethered particle was recorded for two seconds, and the oscillation profile exhibits stepwise changes over time due to the binding or unbinding of DNA. The change occurs at the troughs of the time trace of the oscillation (indicated by the red dashed line) where the particle is at the maximum distance

from the surface. The switching in the DNA tethers has an impact on the determination of the phase. We extracted the phase for the same particle at different tether numbers and observed variation in the phase (Figure S2b).

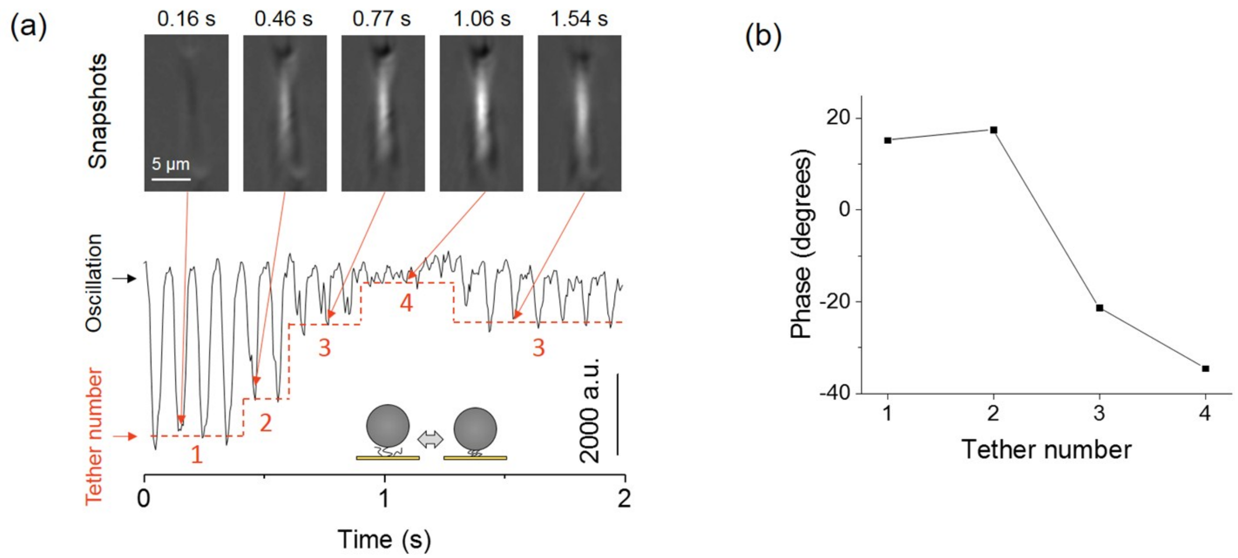

Figure S2. Switching in the number of DNA tethers. (a) Top panel: Snapshots of the plasmonic images of an oscillating particle at the time points. Bottom panel: Oscillation of the particle, where the red dashed line indicates the change of oscillation amplitude associated with the switching of DNA tethers. (b) Phases of the particle with 1-4 DNA tethers were obtained by performing FFT on the image sequences recorded in 0-0.4 s, 0.4-0.6 s, 1.4-2 s, and 0.9-1.2 s, respectively. Experimental conditions: 5 μm silica particle tethered to a gold surface with 500 nm DNA molecules and driven into oscillation with a potential of 500 mV (vs. Ag/AgCl) at 10 Hz. The images are recorded at 200 frames per second.

Phases of particles with multiple DNA tethers

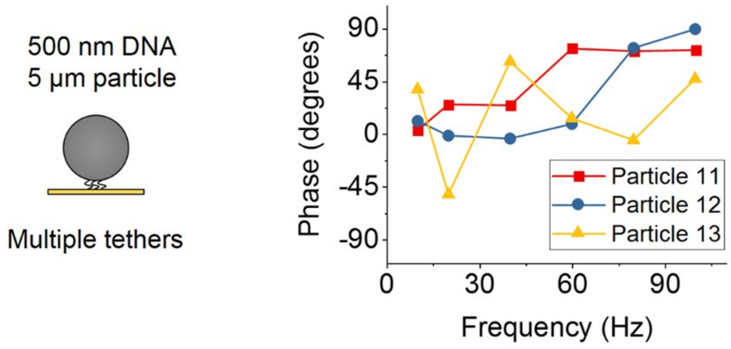

Figure S3. Phases of particles tethered by multiple DNA molecules. Phases of three particles tethered by multiple (at least two) DNA molecules vs. frequency. Unlike the phase of single DNA tethered particles, the phase for multiple DNA tethered particles show large variability and cannot fit well to Eq. 2.

Particle-to-particle variability and assay-to assay variability

The particle-to-particle variability of 5 um silica particles are shown in Figure S4a, where the phase distribution for each frequency is measured from ~50 individual particles (Table S1). We obtain mean values and standard deviations from the phase distributions and fit the phase vs. frequency plot to Eq. 2 (Figure S4b). The spring constant k is determined to be $(4.2\pm3.1)\times10^{-6}$ N/m and the R value (with error) at different frequencies are plotted in the bottom panel in Figure S4b.

The assay-to-assay variability is investigated by measuring the phase of the same 5 µm particle for 200 times. The standard deviation in phase is 0.47°, which is much smaller than the particle-to particle variability. Therefore, we conclude that our phase measurements are accurate and the variability in phase is due to different particles.

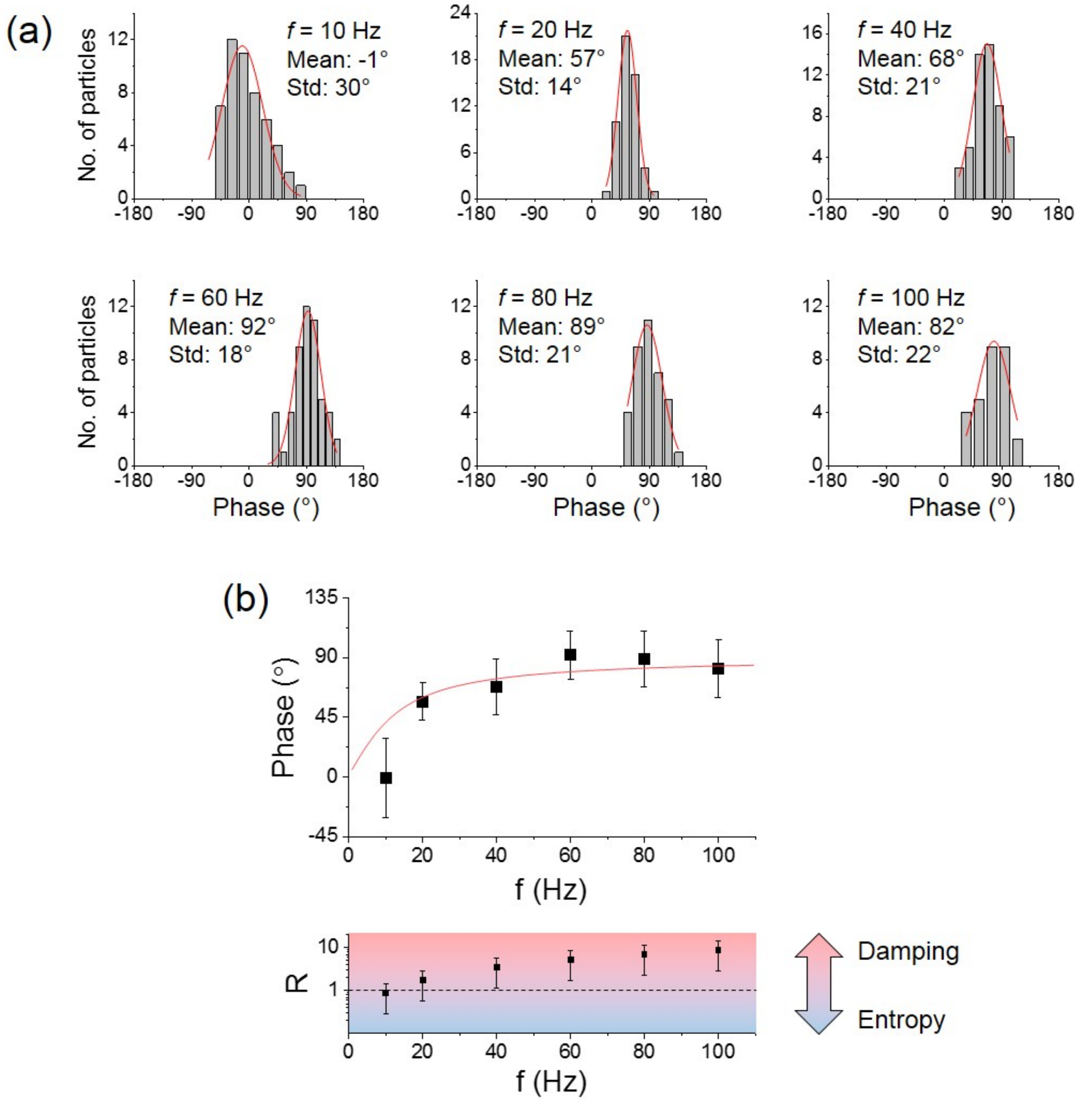

Figure S4. Phase distributions of particles at different frequencies. (a) Histograms of phase distributions of individual particles at 10 Hz, 20 Hz, 40 Hz, 60 Hz, 80 Hz and 100 Hz, respectively. The red curves are Gaussian fittings to the distributions. Each particle (size, 5 μm) is tethered by a single DNA molecule (length, 500 nm). The mean value and standard deviation (Std) are indicated for each distribution. (b) Top panel: The oscillation phase obtained in (a) is plotted vs. frequency, where the solid curve is fitting of the data to Eq. 2. The entropic spring constant is determined to be $(4.2\pm3.1)\times10^{-6}$ N/m. Bottom panel: Relative importance of damping and entropy at different frequency. The phases in the top panel are converted to *R*

values using Eq. 2 and $R = \frac{\omega c}{k}$. The dashed line marks $R = 1$, where damping and entropy are equally important.
